# Mitochondrial and glycolytic extracellular flux analysis optimization for isolated pig intestinal epithelial cells

**DOI:** 10.1038/s41598-021-99460-0

**Published:** 2021-10-07

**Authors:** A. F. Bekebrede, J. Keijer, W. J. J. Gerrits, V. C. J. de Boer

**Affiliations:** 1 Human and Animal Physiology, Wageningen University and Research, 6708 WD Wageningen, The Netherlands; 2 Animal Nutrition Group, Wageningen University and Research, 6708 WD Wageningen, The Netherlands

**Keywords:** Metabolism, Animal biotechnology

## Abstract

Intestinal epithelial cells (IECs) are crucial to maintain intestinal function and the barrier against the outside world. To support their function they rely on energy production, and failure to produce enough energy can lead to IEC malfunction and thus decrease intestinal barrier function. However, IEC metabolic function is not often used as an outcome parameter in intervention studies, perhaps because of the lack of available methods. We therefore developed a method to isolate viable IECs, suitable to faithfully measure their metabolic function by determining extracellular glycolytic and mitochondrial flux. First, various methods were assessed to obtain viable IECs. We then adapted a previously in-house generated image-analysis algorithm to quantify the amount of seeded IECs. Correcting basal respiration data of a group of piglets using this algorithm reduced the variation, showing that this algorithm allows for more accurate analysis of metabolic function. We found that delay in metabolic analysis after IEC isolation decreases their metabolic function and should therefore be prevented. The presence of antibiotics during isolation and metabolic assessment also decreased the metabolic function of IECs. Finally, we found that primary pig IECs did not respond to Oligomycin, a drug that inhibits complex V of the electron transport chain, which may be because of the presence of drug exporters. A method was established to faithfully measure extracellular glycolytic and mitochondrial flux of pig primary IECs. This tool is suitable to gain a better understanding of how interventions affect IEC metabolic function.

## Introduction

The intestine forms a physical barrier against the outside milieu. Multiple factors can impair intestinal barrier function, of which the most common ones are stress^1,2^, infectious agents^3,4^ and drug use^5–7^. If the barrier fails, this can lead to intestinal problems such as diarrhea. Diarrheal diseases are a serious health concern, especially in third world countries^8^ and in animal husbandry^9,10^. Diarrhea in early life can have long lasting effects; the malnutrition it causes has been shown to increase the susceptibility for developing metabolic disorders in later life^11^. Intestinal epithelial cells have a central role in maintaining the intestinal barrier function, for which they rely on energy supply by mitochondria and glycolysis.

Intestinal epithelial cell (IEC) energy metabolism, and especially mitochondrial energy production, is essential for maintaining the intestinal barrier function both in vitro and in vivo^6,12,13^. Tight junction proteins that are responsible for tethering the IECs together rely on sufficient energy generation, since inhibition of mitochondrial ATP production internalized the tight junction protein claudin 7 because of an energy crises, resulting in a loss of barrier^12^. Also other intestinal functions, such as nutrient digestion, uptake and metabolism, rely on energy generation by the mitochondria. After a meal, when nutrient processing is highly upregulated, small intestinal oxygen consumption doubles to satisfy energy needs^14^. In the colon, IECs are faced with additional metabolic challenges, since IECs are then in constant contact with the microbiome. For example, bacteria target and inhibit mitochondrial function of colonocytes to increase their virulence^3,4^, which decreases intestinal barrier function and promotes bacterial translocation^15^. Thus, IEC metabolic function is important for maintenance of intestinal barrier function, as well as the response to external and internal stressors.

Assessment of intestinal metabolic function in both animal and human in vivo experiments is not often considered as an outcome parameter of interventions that target intestinal function. Part of this hiatus is caused by difficulties in measuring intestinal epithelial energy metabolism, especially mitochondrial respiration and the enterocyte glycolytic flux. Measurement of IEC metabolism is extra challenging because of the high turnover rate of the intestinal epithelium of every 5–7 days^16^, which can indicate that the fully differentiated enterocytes only have a short life-span, and isolation of these cells likely rapidly leads to apoptosis^17^. We set out to design a procedure to isolate intestinal epithelial cells and characterize their metabolism. We chose to optimize the isolation procedure for the colon, because microbial fermentation end-products present in this intestinal compartment can both positively and negatively impact mitochondrial function of IECs. For example, butyrate, a short-chain fatty acid produced through fibre fermentation, is known to be an important energy source for colon cells and thus supports mitochondrial function^18,19^. On the other hand, hydrogen sulfide, which is produced through protein fermentation, decreases mitochondrial function^20^. We use the pig both as a model and a relevant target species. Pigs are an important agricultural species^21^, and are also the best human translatable animal model to study large organs systems, such as the intestinal tract^22^. Apart from the high similarity between pigs and humans in GI tract anatomy, a great advantage for using pigs as a model lies in the similar clinical manifestations and their susceptibility to many enteric pathogens and intestinal diseases afflicting humans^23,24^. Other commonly used animal models like mice are less similar to humans than pigs with regard to their intestinal metabolism, microbial pathways and their response to nutritional interventions^24–26^. A more practical consideration for using pigs, is the availability of adequate amounts of healthy intestinal material.

Here, we designed an IEC isolation technique to harvest viable primary IECs from the pig colon, that is suitable for measurement of mitochondrial respiration and glycolytic flux. We optimized the flux analysis for chemical induced signal stability and normalization procedures for cell number correction. Our results showed that the optimized cell isolation technique was suitable for measurement of metabolic function of pig IECs. This technique will be a useful tool to evaluate the effect of interventions on intestinal function.

## Results

### Pig primary IEC isolation optimization for analyzing metabolic activity

**Figure 1:**
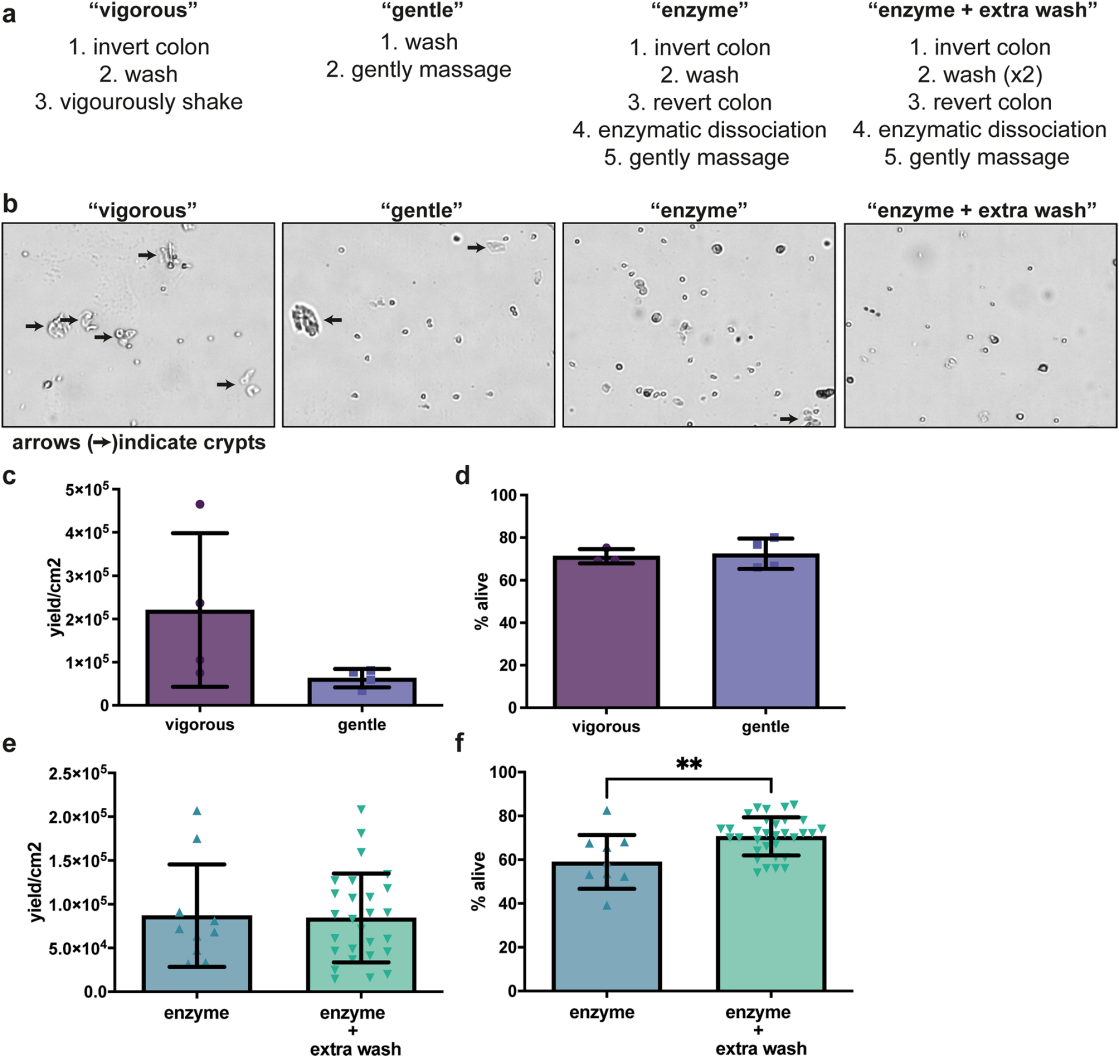
Comparison and optimization of enterocyte isolation methods. (**a**) A brief overview of the experimental procedures of for the different isolation methods used during the optimization procedure. (**b**) Representative brightfield images of the isolated cell populations for the different isolation methods; crypts are indicated with arrows. (**c**,**d**) Analysis of yield and viability of the vigorous and gentle isolation methods (n = 4 pigs per isolation method). (**e,f**) Analysis of yield and viability of enzymatic (enzyme) and enzymatic with additional washing step (enzyme + extra wash) dissociation methods (n = 8 pigs for enzyme procedure, n = 27 pigs for enzyme + extra wash). Student’s t-tests were performed to compare yield and viability of the enzyme and enzyme + wash methods, **indicates a *p*-value of ≤ 0.01. Each dot in the bar-graphs represents an IEC isolation from a single pig.

We set out a strategy to set-up our methodology using a number of shearing and isolation methods described in literature (Fig. 1a). For reference, we compared the methods described by Roedinger and Truelove^27^ and Darcy-Vrillon et al.^19^. The former used vigorous shaking while the latter used gentle massaging of the intestinal segments, and we will thus refer to them as “vigorous” and “gentle”, respectively. Both methods were performed in physiological buffer without enzymatic treatment. To be able to compare methods, we first scored the presence of crypts in the isolates as a sign of isolation robustness. More crypts means that less single cells are isolated, which makes the method less suitable. The vigorous as well as the gentle method both gave crypts (Fig. 1b), although slightly less in the gentle method, and the overall yield and viability was not different between both techniques (Fig. 1c,d). Although viability was not different, basal OCR was significantly lower for the vigorous method (supplementary Fig. S1a). Therefore, we decided to further optimized the gentle method, by inverting the intestine during the washing steps to increase the exchange between buffer and intestinal surface area. In addition, we added an enzymatic dissociation step in order to improve single-cell yield (‘enzyme’ method). After including these steps, we observed less crypts compared to the method without inversion and enzymatic dissociation (Fig. 1b). Next, to be able to isolate IECs from piglets that received different feeds that could possibly affect the thickness of the mucus layer, we added an extra washing step to the isolation protocol to increase mucus removal prior to enzymatic cell dissociation (‘enzyme + wash’ method; final protocols see supplemental method for a stepwise description). Introduction of the extra washing step did not result in lower cell yield (Fig. 1e), and increased cell viability (Fig. 1f, *p* = 0.0018), demonstrating that extra washing can be included without negatively impacting cell viability and yield, but allowing for a flexible isolation protocol based on the feeding status of the animals. In the subsequent experiments, the ‘enzyme” and “enzyme + extra wash” methods were used, unless otherwise stated.

### Optimization of normalization methodology for pig IEC metabolic measurements

**Figure 2:**
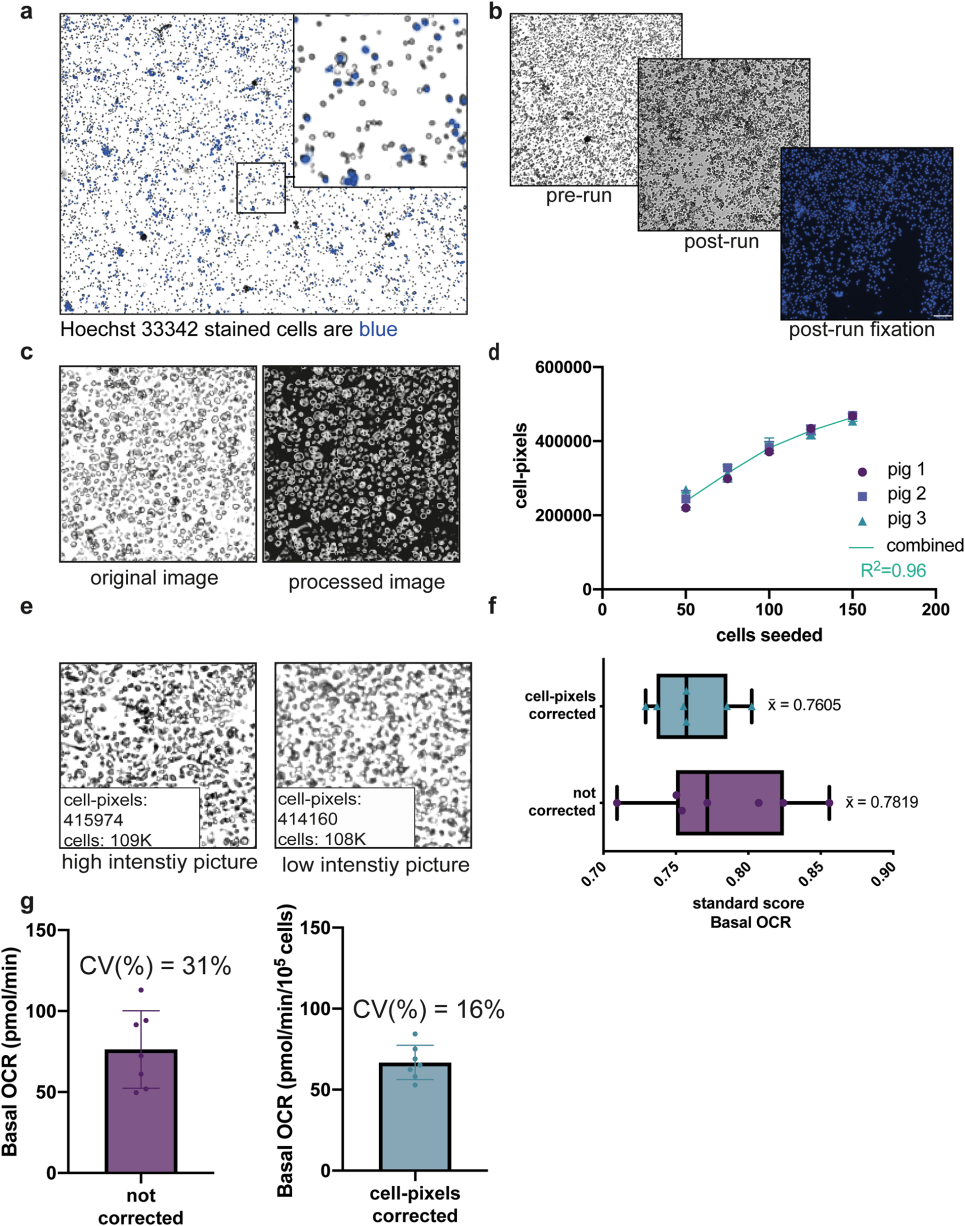
Optimization of normalization for metabolic Seahorse measurements. (**a**) Hoechst 33342 staining of isolated IECs. (**b**) Images depicting the same well pre-run, post-run and post-fixation and DAPI staining. (**c**) Pre-run brightfield images are processed for image analysis, yielding a white-object-on-black-background image. (**d**) Correlation of cell-pixels to the number of cells expected to be seeded for three pigs and their combined correlation coefficient (50,000–150,000 cells/well, n = 4 wells per cell concentration). The combined calibration curve for the three pigs was used to convert cell-pixel values back into cell numbers. (**e**) Images with varying image intensities show similar cell-pixel values, with corresponding also similar cell number values. (**f**) Within-pig variation, as represented by standard score, of basal OCR measurement in a group of piglets corrected using our cell-pixel image analysis (n = 7 pigs). (**g**) Coefficient of variation (%), as a measure of between-pig variation, of basal OCR measurement in the same group of piglets (n = 7). Representative images are shown.

Next, we analyzed extracellular O_2_ (OCR) and pH (ECAR) fluxes of primary isolated pig IECs using the Seahorse XFe96 analyzer. This allows for simultaneous measurement of oxygen consumption, as a measure for mitochondrial respiration, and extracellular acidification, as a measure for glycolytic function. An important step in this analysis is adjustment of the data to the actual cell number in the well, which can fluctuate despite cell counting prior to the addition of the cells to the plate. We therefore optimized the normalization method. First, we tested nuclear staining with Hoechst, but noticed that not all cells were stained, as can be observed from the non-blue stained cells present in the picture (Fig. 2a). This was likely due to active efflux drug transporters in the IECs^28^. Next, we fixed the cells in wells and stained them with DAPI. Because the IECs were only loosely attached to the assay plate, and fixation and DAPI staining required several washing steps, cells were often washed away after the procedure (Fig. 2b). Next, we used brightfield (BF) images of the wells, obtained prior to the Seahorse XF analysis, similar to a method performed for primary PBMCs^29^ (Fig. 2c). We observed a correlation coefficient of 0.95–0.99 between the number of cells we seeded from three pigs and the number of cell-pixels counted using our R-algorithm (Fig. 2d). A drawback of this correction method is that there can be inter-plate differences in image acquisition, necessitating the addition of a standard curve on each plate^29^. However, we observed that differences in image intensity did not result in different amounts of counted cells, making the use of in-plate calibration curve unnecessary for our experimental set-up and cell type (Fig. 2e). We combined the data of the three piglets (Fig. 2d) and performed a second order polynomial fit analysis, which we used as a calibration curve. Following transformation of pixel intensities to cell number using the formula obtained from the combined data of the three pigs (R^2^ = 0.96), we applied this normalization method to a group of piglets (n = 7) that received an experimental diet for two weeks. We found that normalization indeed reduced the within subject variation of basal OCR values, as can be analyzed using the average z-score (or standard score, see Eq. (1) for calculation; Fig. 2f). The standard score represents the variation within the technical replicates (n = 4–10 wells/pig) that are included during the Seahorse XF analysis. The arithmetic mean of the standard score decreased from 0.78 to 0.76 after correction for cell number and the range of standard scores was smaller (Fig. 2f). The coefficient of variation (CV), which represent the between pig variation, decreased from 31 to 15% (Fig. 2g). These results indicate that normalization using bright field imaging for pig IEC Seahorse analysis lowers both within and between subject variation, which benefits statistical interpretation and the number of replicates that are needed.

### Optimization of metabolic analysis using isolated pig primary IECs

**Figure 3:**
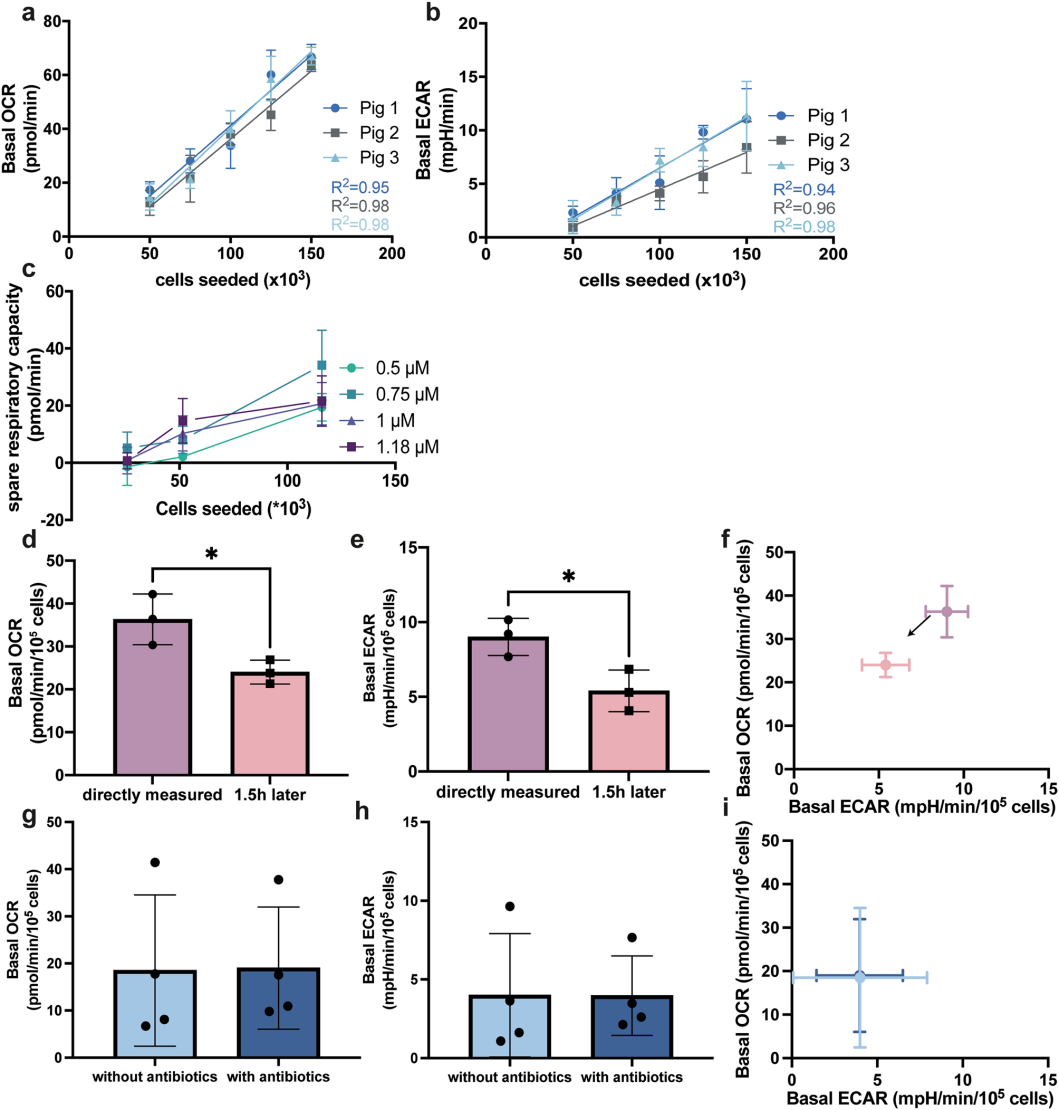
Optimization of Seahorse metabolic flux analysis of isolated pig IECs. (**a**,**b**) Comparison of the linear correlations between basal OCR and basal ECAR versus seeded cells, in a range of 50,000–150,000 cells (n = 4 wells per concentration). (**c**) Optimization of FCCP concentration for different cell concentrations, ranging from 25,000 to 115,000 cells/well (n = 3–5 wells per concentration, cells were isolated using the ‘gentle’ method). (**d**–**f**) Effect of leaving IECs in the plate for 1.5 h on their basal OCR, basal ECAR and energetic phenotype (n = 3 pigs). (**g**–**i**) Effect of addition of antibiotics to the isolation buffers and assay medium of IECs on their basal OCR, basal ECAR and energetic phenotype (n = 4 pigs with and n = 4 pigs without antibiotics). One-way ANOVA was performed to identify the optimal FCCP concentration, while student’s t-tests were performed to compare OCR and ECAR after leaving cells in the plate for 1.5 h and the effect of antibiotics, *indicates a *p*-value of ≤ 0.05.

After establishing the proper strategy to correct for cell number, we determined the number of cells needed to obtain a sufficient signal during a Seahorse run. We observed a strong correlation between the number of seeded cells and basal OCR (Fig. 3a) as well as basal ECAR when cells were seeded within a range of 50,000–150,000 cells per well (Fig. 3b), showing that this range was suitable for analysis. Spare respiratory capacity is an important metabolic parameter that often can distinguish metabolic states between cell populations. Therefore, we studied the optimal FCCP concentration needed for uncoupling. Both too low and too high concentrations of this drug will result in sub-optimal uncoupling. We noticed that at a low cell seeding density (25,000 cells per well) spare respiratory capacity (SRC) was not detectable. At 50,000 cells per well and at 120,000 cells per well a different concentration FCCP was observed to induce the highest SRC. For 50,000 cells per well 1.18 µM FCCP induced the highest increase in respiration, while this was 0.75 µM FCCP at 115,000 cells per well, although there were no significant differences between the different FCCP concentrations (Fig. 3c). To achieve experimental uniformity, we decided to continue our experiments using 100,000 cells per well and 1 µM FCCP.

Given that isolated primary IECs do not remain viable for extended periods of time, we evaluated the effect of delaying metabolic function analysis by leaving cells in the plate after isolation. We observed that leaving the cells in the plate for 1.5 h prior to metabolic measurement resulted in a significant decrease of basal OCR as well as ECAR, compared to immediate measurement (Fig. 3d,e). In Fig. 3f, when plotting OCR and ECAR in an energetic phenotype plot, it can be seen that a delay of 1.5 h already resulted in movement of the energetic phenotype towards the less energetic quadrant. These results indicated that the metabolic function should be analyzed as soon as possible after isolation and at least at a defined time interval smaller than 1.5 h.

Microbial contamination of the isolated cell population can disturb the metabolic measurements of isolated cells. Therefore, antibiotics have been used in enterocyte isolations to lower the risk of contamination. However, antibiotics have also been shown to alter cellular metabolism^30,31^. We therefore investigated the effect of isolation of cells in the absence or presence of 1% (v/v) penicillin/streptomycin. We did not observe differences in basal OCR and ECAR of intestinal cells isolated in the presence of antibiotics as compared to absence of antibiotics (Fig. 3g–i). Even in absence of antibiotics, bacterial contamination is unlikely to contribute substantially to basal OCR and ECAR, because bacteria are much less dense than cells and it is thus likely that during spin-down at low g-force most of the bacteria are still in the supernatant fraction. In addition, the bacteria need to be attached to the bottom of the plate and be present in the small transient measurement chamber during XF analysis to contribute to the OCR and ECAR, which is likely not the case. To show that the OCR we measure is not due to bacterial contamination, we collected the supernatant after the final wash-step and added this to the plate. The gentle spin resulted in some cells being present in the supernatant fraction (supplementary Figure S2a). However, OCR levels of supernatants were lower than the 20 pmol/min detection limit that is typically used as a cut-off for reliable Seahorse OCR measurements (supplementary Figure S2b), whereas the cells had OCR levels ranging from 28 to 110 pmol/min (supplementary Figure S2b). Thus, the OCR that is measured cannot originate from bacterial contamination, but is instead associated with the intestinal cellular fraction. Therefore, we decided not to use antibiotics and practiced robust sterile working conditions and proper washing of cell isolates.

**Figure 4:**
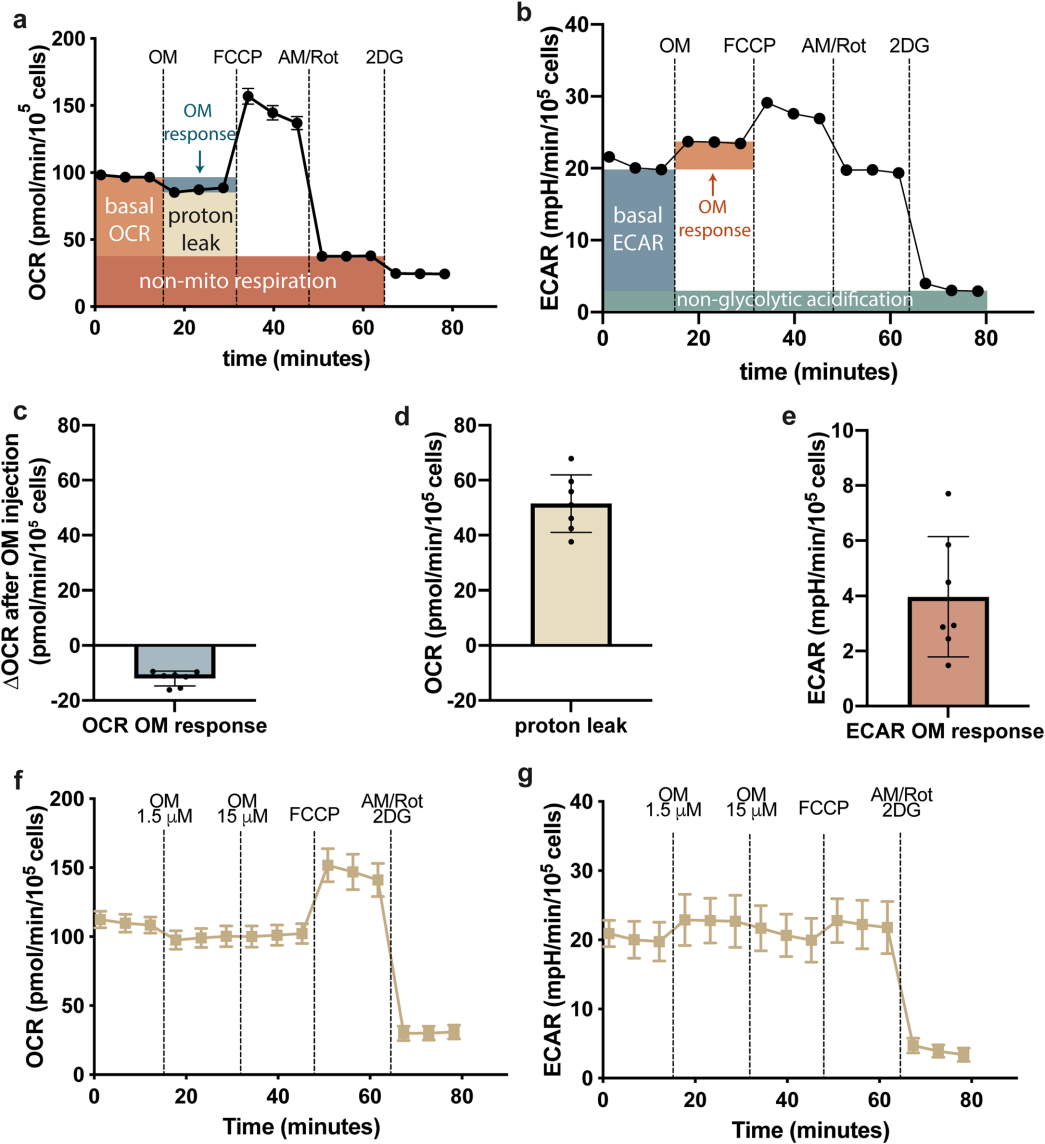
Effect of Oligomycin on oxidative and glycolytic function of isolated IECs. (**a**,**b**) The average time-course trace for OCR and ECAR of n = 7 pigs, following subsequent injections of Oligomycin, FCCP, Antimycin A, Rotenone and 2-DG, depicting which time-points are used for the calculations. (**c**) Change in OCR of isolated pig IECs after Oligomycin injection (n = 7). (**d**) Calculated proton leak of isolated pig IECs (n = 7). (**e**) Effect of Oligomycin on ECAR in pig IECs (n = 7). (**f**,**g**) Time-course traces of OCR and ECAR, showing the effect of addition of a ten-fold higher Oligomycin concentration (average traces of n = 6 pigs).

During XF analysis, Antimycin A and Rotenone are used to attain maximal inhibition of mitochondrial respiration by respectively blocking complex III and I of the electron transport chain. We have titrated AM/Rot for several cell lines in the past and always obtained maximal inhibition with the doses we use here. During analysis of IEC metabolic flux analysis, we observed an additional decreased in OCR following addition of 2DG (Fig. 4a). This additional decrease could be due to the presence of non-mitochondrial oxidases, that may respond physiologically to inhibition of glycolysis by injection of 2DG. Some of the known processes that can contribute to non-mitochondrial respiration are NADPH oxidases and even electron cycling at the plasma membrane^32^, which can be dependent on glycolytic metabolism to generate NADPH or NADH substrates, and are therefore inhibited by 2DG.

Apart from FCCP, Antimycin A and Rotenone, the drug Oligomycin, a blocker of F_0_F_1_-ATPase (Complex V) is used to assess mitochondrial respiration and glycolytic flux. Typically, Oligomycin lowers OCR and increases ECAR substantially as a response of the cells to compensate for loss of mitochondrial ATP production. However, the average OCR and ECAR time-course traces of primary IECs show only a slight effect of 1.5 µM OM (Fig. 4a,b). Indeed, we observed only a limited decrease in oxidative respiration, and a concomitant limited increase in glycolytic function (Fig. 4c,e). As a result, the isolated IECs seem to have a relatively high proton leak (Fig. 4d). We investigated whether the unresponsiveness to Oligomycin could be due to the enzyme used during the dissociation method, but found that IECs isolated using the non-enzymatic ‘gentle’ isolation method also did not respond to 1.5 µM Oligomycin (supplementary Fig. S3a). To rule out that unresponsiveness to Oligomcyin could be due to possible bacterial contamination, we investigated the response to Oligomycin in the presence and absence of antibiotics. If bacterial contamination indeed contributes to the lack of response of Oligomycin, antibiotics treated cells should show an Oligomycin response. However, we did not observe a significant difference between Oligomycin inhibition of cells isolated with and without antibiotics (supplementary Fig. S3b). Another explanation was that the Oligomycin is unable to properly block complex V activity, which has also been observed for other cell types^33–35^. Indeed, increasing the Oligomycin concentration tenfold did not elicit an additional response in OCR and ECAR, indicating that Oligomycin is likely not able to properly inhibit complex V in isolated primary pig IECs (Fig. 4f,g).

## Discussion

Intestinal barrier function is decreased when energy production is impaired, which can occur through inhibition by drugs, redirection of blood flow during strenuous exercise or even upon bacterial infection^3,6,13^. Thus, IEC metabolism plays an important role in supporting intestinal barrier function. However, there is a lack of availability of good research protocols to study IEC metabolism. In this paper we successfully developed a method to isolate primary pig IECs and optimized the analysis of their energy metabolism. The method yielded a population of viable, single cells and can be easily adjusted to facilitate e.g. better mucus removal by altering the number of washing steps, without decreasing cell viability. In addition, the technique can be applied to a wide range of pig ages, sexes and breeds, as we have demonstrated by using a mix of ages, sexes and breeds throughout the optimization procedure. We also adapted an algorithm to normalize the Seahorse XF data for the number of cells in the assay using brightfield images obtained before the run, which reduced the coefficient of variation of basal respiration between the pig IEC isolates. Furthermore, our data showed that delay of measurement after IEC isolation as well as the use of antibiotics negatively impact the metabolic function of the primary IECs. Measurement immediately after isolation is advised. Combined with optimization of cell densities, medium and chemical composition and concentrations, we have obtained a robust procedure to measure metabolic function of primary isolated pig IECs using the Seahorse Extracellular Flux analyzer.

The method described in this paper will improve our understanding on the role of mitochondrial function in intestinal health in pigs as well as humans. Applying this method can be especially interesting for studies with interventions that alter the luminal environment of the intestine, such as dietary interventions. Because such interventions can be performed in vivo*,* this enables all the complex interactions within the intestinal environment to take place. Currently, our knowledge on the impact of dietary interventions on intestinal epithelial cell metabolic function is limited. There are some reports of how dietary interventions, such as high fiber diets^19^, can affect intestinal mitochondrial function, but are mostly limited to oxygen consumption analysis. The use of Seahorse Extracellular Flux analysis facilitates simultaneous measurement of oxygen consumption and pH change, and thus allows for the simultaneous assessment of oxidative and glycolytic metabolism. Our isolation technique mainly results in the isolation of fully differentiated IECs, which have a metabolically active phenotype, with both high mitochondrial as well as glycolytic function^36,37^. It is therefore important to include both these pathways in metabolic analysis of intestinal epithelial cells. It will be interesting to investigate whether interventions that reduce mitochondrial oxidative functions also reduce glycolytic function, or if glycolytic function can actually be increased to compensate for the loss of mitochondrial ATP production, as is sometimes observed^38^. A limitation of our study is that we did not normalize our functional data using cell characteristics that could affect the oxygen consumption of the cells. When performing oxygraphic analysis, it is common practice to correct for mitochondrial mass using mitochondrial DNA or Citrate Synthase. Further development of our method to include such characteristics could be useful to in to future even better understand what underlies the changes in Extracellular Flux data.

In this study, we used primary IECs to investigate the metabolic consequence of antibiotics. In literature, antibiotics were found to inhibit mitochondrial function, increase production of reactive oxygen species, disrupt mitochondrial biogenesis and induce mitochondrial-mediated apoptosis in cancer cells^39,40^. Specifically, antibiotics were found to reduce the expression of the respiratory chain complexes in mouse ileal tissue^41^. Also in cultured cell lines, including those derived from the intestine, antibiotics were found to decrease the function of mainly complex I and III of the electron transport chain, with a concomitant increase in reactive oxygen species (ROS) production^31,42,43^. Antibiotics are typically used routinely in cell culture, even when those cells are later used for metabolic assessments. In some IEC isolation procedures, antibiotics are also added to the isolation medium, which is intended to reduce bacterial contamination^19,44^. This, however, may not be ideal if subsequent metabolic parameters are analyzed. Since mitochondrial function impacts a wide array of cellular functions^14,45^, including barrier function^12^, the use of antibiotics will likely also affect other processes. Therefore, we do not recommend the use of antibiotics during isolation of IECs, even though our results show no direct effect of antibiotics on cellular intestinal metabolism fluxes (Fig. 3g–i). In addition, the lack of effect of antibiotics on OCR and ECAR and the low OCR and ECAR measured in the supernatants (supplementary Fig. S2) also indicates that bacterial contamination does not significantly contribute to the OCR and ECAR that we measure. Thus, antibiotic use may not be necessary to accurately measure metabolic function of isolated IECs. Inclusion of additional wash steps in the IEC isolation procedure may help to reduce bacterial contamination and permits the omission of antibiotics during the isolation procedure. We showed that more washing steps can be included, e.g. if the mucus layer is thick, without affecting cell viability and yield (Fig. 1e,f).

During analysis of isolated pig IEC metabolism we observed a relatively small change in OCR and ECAR upon oligomycin injection (Fig. 4). A possible reason for a lower responsiveness of our cells to Oligomycin may be the presence of active efflux drug transporter in these primary IECs^28^. Oligomycin analogues have been shown to inhibit P-glycoprotein mediated calcein-AM transport, indicating that these oligomycin analogues interact with these efflux transporters^46^. Furthermore, it has also been shown that the mitochondrial ATPase enzyme itself can be less sensitive to Oligomycin^33^. Mutations in complex V have been shown to block Oligomycin binding^34,47^, but without compromising proton translocation^34^, indicating that unresponsiveness to Oligomycin is not always paralleled by uncoupled respiration. Also certain pig cells, boar sperm, were found to be insensitive to Oligomycin with regard to decreases in ATP production and oxygen consumption^35^. Interestingly, Oligomycin did result in decreased sperm motility, indicating that Oligomycin probably caused off-target effects in these cells^35^. With regard to IECs specifically, a inhibitory effect of Oligomycin has been reported for the pig-derived cell line IPEC-J2^48^. However, these are cultured cells that are in long-time culture, and have possibly accumulated mutations which can affect their metabolic responses. In mice, Fan et al*.* also observed a small increase in ECAR in response to Oligomycin and reported that the oxygen used for ATP production was half of that contributing to the proton leak in isolated mouse colonic crypts^49^, which is in line with our observed Oligomycin responses.

In conclusion, we have successfully developed a method to isolate viable pig primary IECs and faithfully measure their extracellular glycolytic and mitochondrial flux. As a whole, the method we present may likely be a useful tool to be included for functional analysis of the effects of various interventions on intestinal health, thus providing new insight into the complex interactions in the intestinal environment; the intestine that is simultaneously the gateway and the gatekeeper towards the rest of the body.

## Methods

### Animals

Intestines were either harvested from pigs at a slaughterhouse or from control pigs from dedicated animal experiments sacrificed at our Animal Facility, that were all approved by the Animal Care and Use Committee of Wageningen University. However no animals were sacrificed specifically for the purpose of this study. The majority of material was derived from slaughterhouses. We were unable to get all details for pig breeds, age and sex, because we choose for a rapid, relatively easy organ collection procedure instead of a detailed dissection. This offers ease in performing multiple experiments on scheduled cell isolation days, and shows the flexibility of the isolation procedure. Overall, the pigs were from both sexes, multiple breeds, weighed between 20 and 100 kg and were aged between 12 and 32 weeks.

### IEC isolation

Following excision from the abdominal cavity, an approximately 20 cm long segment of the colon was taken for IEC isolation and placed in aerated Krebs Henseleit Buffer containing 5 mM glucose (#K3753, Sigma Aldrich; hereafter referred to as modified-KHB), containing 2.5 g/L Bovine serum albumin (BSA, #A7906, Sigma-Aldrich). Samples were transferred to the lab and isolation commenced within 2 h after killing the animal.

Multiple steps were taken to optimize the isolation procedure. Initially, two methods described in literature were used as a basis for the design of the procedure. First, a method described by Roedinger and Truelove^27^ was assessed. We refer to this method as the vigorous method. The colon segments were first flushed with room temperature (RT) modified-KHB, inverted and then a sac was created using dialysis clamps (#Z371092, Sigma Aldrich) and the sac was filled with modified-KHB. The sacs were then placed in Ca^2+^-free KHB with 5 mM Ethylenediaminetetra-aceticacetic acid (EDTA) and 2.5 g/L BSA. After a 30-min incubation in a shaking 37 °C water bath, the buffer was removed and replaced by fresh Ca^2+^-free KHB containing 2.5 g/L BSA. The intestines were stirred vigorously by hand for two minutes to dissociate the IECs. IECs were then passed over a 70 µM cellulose filter top to remove large tissue pieces and debris. After washing cells twice using modified-KHB contained 2.5 g/L BSA, cells were taken up in pH 7.4 buffered XF DMEM medium (#103575-100, Agilent Technologies) supplemented with 10 mM glucose (#103577-100, Agilent Technologies), 2 mM glutamine (#103579-100, Agilent Technologies) and 1 mM pyruvate (#103578-100, Agilent Technologies) and counted using a Cellometer K4 (Nexcelom Bioscience) and viability was simultaneously assessed by staining with ViaStain (#CS2-0106, Nexcelom Bioscience). The second method tested, to which we refer as the gentle method, was a modification of the one described by Darcy-Vrillon et al.^19^. In this method, the intestine was first flushed with modified-KHB, and then immediately a sac was created using dialysis clamps. The sac was filled with Ca^2+^-free KHB containing 10 mM EDTA, 5 mM Dithiothreitol (DTT), and 2.5 /L BSA. After a 20-min incubation in a shaking 37 °C water bath, the sac was emptied and refilled with the same buffer, followed by another fifteen-minute incubation. Afterwards, the intestines were gently massaged, and cells were collected, washed and counted, as described for the first procedure.

The third method we tested (which was also the optimized method we used for downstream analysis of metabolic function) was a combination of steps from the above two methods combined with a hyaluronidase enzymatic dissociation step and optional washing steps. We refer to this method as the ‘enzyme’ method or ‘enzyme + extra wash’ method (see supplementary materials for a stepwise lab protocol of the final optimized method). First, intestines were flushed with modified-KHB. Then, they were inverted, and a sac was created using dialysis clamps by filling them with modified-KHB. Inversion of the intestines at this stage facilitated exchange between buffer and mucosa, since the amount of buffer can be much higher than if the intestines are not inverted. The sacs were first incubated for 20 min in Ca^2+^-free KHB buffer containing 20 mM EDTA and 10 mM DTT in a shaking 37 °C water bath. Following this washing step, intestines were re-verted and filled with an isolation buffer containing Ca^2+^-free KHB buffer, 2.5 g/L BSA and 400 U/mL hyaluronidase type IV (#3884, Sigma-Aldrich), an enzyme that catalyzes the breakdown of hyaluronic acid which is present in the extracellular matrix of IECs. The re-version of the sacs at this stage is convenient, since cells will be collected in a smaller volume. In addition, the amount of buffer and enzyme needed can thus be reduced, which is cost-effective. After a fifteen-minute incubation, the intestines were gently massaged and cells were collected, washed and counted as previously described. This protocol was finally adjusted by adding a 20 min washing step before the enzymatic digestion to facilitate increased removal of mucus from the intestines.

For the experiments where we analyzed the effects of antibiotics on metabolic function of IECs, all washing and isolation buffers used in the isolation procedure were supplemented with 1% v/v penicillin–streptomycin (#15140122, Fisher Scientific).

### Metabolic flux analysis with seahorse XFe96 analyzer

Isolated IECs were plated in a XF96 cell plates that were coated with Cell-Tak (#354240, Corning, New York, USA) according to manufacturer’s protocol, no longer than one week prior to the assay. Cells were plated at concentrations ranging from 25,000 to 150,000 cells/well in 50 µL pH 7.4 balanced XF DMEM assay medium supplemented with 10 mM XF glucose, 2 mM XF glutamine and 1 mM XF pyruvate. For the normalization optimization and Oligomycin response experiments, cells were plated at 100,000 cells/well, left to settle for 5 min prior to spin-down (200 × *g* for 2’ with zero break). After spin-down, cell plates were imaged as described below, while kept at 37 °C. Following imaging, an additional volume of 130 µL assay medium was added and cell plates were incubated for another 20 min in a non-CO_2_ 37 °C incubator. For the optimization of carbonyl cyanide-p-trifluoromethoxyphenylhydrazone (FCCP; #C2920, Sigma-Aldrich), cells were isolated using the ‘gentle’ method and final concentrations of 0.5–1.18 µM were injected into the wells. For optimization of Oligomycin (a mix of A, B and C Oligomycin, #O4875, Sigma-Aldrich) concentration, a final concentration of either 1.5 or 15 µM was used. Extracellular flux analyses (XF assays) was performed using the Seahorse XFe96 (Seahorse Bioscience, Agilent Technologies, Santa Clara, USA). Most often, XF assays were performed using serial injections of 1.5 µM Oligomycin, 1 µM FCCP, a combination of 1.25 µM Rotenone and 2.5 µM Antimycin A and finally 50 mM 2-deoxyglucose (2-DG). The XF assay protocol typically consisted of 12 measurement cycles of 3 min, with 2 min of mixing in between measurements. For the measurements with antibiotics, assay medium also contained 1% v/v penicillin–streptomycin. Cell plates were kept in a non-CO_2_ 37 °C incubator for 1.5 h prior to the start of the assay for the delayed measurement experiments.

### Imaging procedure

Brightfield images of the inner probe area of each well in the XF96 cell plates were obtained prior to the XF assay run using a 37 °C equilibrated Cytation 1 Cell Imaging Multi-Mode Reader (BioTek, Winooski, Vermont, USA) using a 4 × objective. A LED intensity of 5 and integration time of 80 ms was kept constant for all cell plates, image focus height was adjusted as needed to get the optimal image quality, as was determined by visual inspection. For optimization of the normalization procedure cell nuclei were either stained using 8 µM Hoechst 33342 (Hoechst, #B2261, Sigma-Aldrich), or fixed using 4% paraformaldehyde (#252549, Sigma-Aldrich) and then stained with 4′,6-diamidino-2-phenylindo (DAPI, #D9564, Sigma-Aldrich), followed by image acquisition using a 4 × objective with a 365 nm LED in combination with an EX337/EM447 filter cube.

### Brightfield image analysis in R

Brightfield images obtained prior to the XF assay run were processed and quantified using an in-house generated R-script that uses the EBImage package available for Bioconductor^50^. Image processing was performed in a similar manner as previously published^29^, with an adjustment of the image quantification. Briefly, a Gaussian blur low-pass filter was applied to generate a background image, followed by subtraction of the background image from the original. The background corrected image was then inverted to generate a “white-objects-on-black-background image”. This image was subsequently cropped by 5% to remove potential noise from the XF assay plate molded stops, that are present on the plates to prevent the sensors from disrupting the cell monolayer. Images were then analyzed to calculate pixel intensity values for all the pixels in the image. All the pixels with an intensity > 1 was counted as representing the presence of a cell, and we refer to these as “cell-pixels”. For conversion of cell-pixels back to cells, an external calibration curve was generated. To do this, a second order polynomial fit analysis was performed on the combined data of three individual pig standard curves. The best-fit curve that matched the data was then used as an external calibration curve to convert cell-pixel values of every plate back to cell numbers. These cell numbers were subsequently used for normalization of the Seahorse XF assays. The R-script is available from GitHub (https://github.com/vcjdeboer/seahorse-data-analysis-PIXI).

### Statistical analysis and data visualization

Data are presented as mean ± s.d., unless stated otherwise. The standard score, or z-score, was calculated using Eq. (1):

1 $${\text{z-score}} = \left( {{\text{well}} - {\text{mean}}\left( {{\text{all wells per subject}}} \right)} \right)/{\text{s.d.(all wells per subject}}).$$

Statistical analyses and data visualizations were performed using GraphPad Prism v.9 (GraphPad Software, CA, USA). Statistical testing was performed using student’s t-test or one-way ANOVA when appropriate and as stated in the figure legends. A *p*-value of < 0.05 was considered statistically significant.

## Supplementary Information

Supplementary Information.

## References

[1] Cao ST (2018). Weaning disrupts intestinal antioxidant status, impairs intestinal barrier and mitochondrial function, and triggers mitophagy in piglets. J. Anim. Sci..

[2] Li Y, Song Z, Kerr KA, Moeser AJ (2017). Chronic social stress in pigs impairs intestinal barrier and nutrient transporter function, and alters neuro-immune mediator and receptor expression. PLoS ONE.

[3] Ma C (2006). Citrobacter rodentium infection causes both mitochondrial dysfunction and intestinal epithelial barrier disruption in vivo: Role of mitochondrial associated protein (Map). Cell. Microbiol..

[4] Josephson H (2020). Pseudomonas aeruginosa N-3-Oxo-dodecanoyl-homoserine lactone impacts mitochondrial networks morphology, energetics, and proteome in host cells. Front. Microbiol..

[5] Mahmud T, Rafi SS, Scott DL, Wrigglesworth JM, Bjarnason I (1996). Nonsteroidal antiinflammatory drugs and uncoupling of mitochondrial oxidative phosphorylation. Arthritis Rheum..

[6] Somasundaram S (2000). Uncoupling of intestinal mitochondrial oxidative phosphorylation and inhibition of cyclooxygenase are required for the development of NSAID-enteropathy in the rat. Aliment. Pharmacol. Ther..

[7] Bjarnason I, Takeuchi K (2009). Intestinal permeability in the pathogenesis of NSAID-induced enteropathy. J. Gastroenterol..

[8] Collaborators GDAI (2020). Global burden of 369 diseases and injuries in 204 countries and territories, 1990–2019: A systematic analysis for the Global Burden of Disease Study 2019. Lancet.

[9] Van Breda LK, Dhungyel OP, Ginn AN, Iredell JR, Ward MP (2017). Pre- and post-weaning scours in southeastern Australia: A survey of 22 commercial pig herds and characterisation of Escherichia coli isolates. PLoS ONE.

[10] Hong TTT, Linh NQ, Ogle B, Lindberg JE (2006). Survey on the prevalence of diarrhoea in pre-weaning piglets and on feeding systems as contributing risk factors in smallholdings in Central Vietnam. Trop. Anim. Health Prod..

[11] DeBoer MD (2013). Early childhood diarrhea and cardiometabolic risk factors in adulthood: The Institute of Nutrition of Central America and Panama Nutritional Supplementation Longitudinal Study. Ann. Epidemiol..

[12] JanssenDuijghuijsen LM (2017). Mitochondrial ATP depletion disrupts caco-2 monolayer integrity and internalizes claudin 7. Front Physiol..

[13] JanssenDuijghuijsen LM (2017). Endurance exercise increases intestinal uptake of the peanut allergen ara h 6 after peanut consumption in humans. Nutrients.

[14] Rolfe DF, Brown GC (1997). Cellular energy utilization and molecular origin of standard metabolic rate in mammals. Physiol. Rev..

[15] Matarrese P (2007). Clostridium difficile Toxin B causes apoptosis in epithelial cells by thrilling mitochondria: Involvement of atp-sensitive mitochondrial potassium channels*. J. Biol. Chem..

[16] Potten CS, Booth C, Pritchard DM (1997). The intestinal epithelial stem cell: The mucosal governor. Int. J. Exp. Pathol..

[17] Grossmann J (1998). New isolation technique to study apoptosis in human intestinal epithelial cells. Am. J. Pathol..

[18] Roediger WE (1982). Utilization of nutrients by isolated epithelial cells of the rat colon. Gastroenterology.

[19] Darcy-Vrillon B (1993). Metabolic characteristics of pig colonocytes after adaptation to a high fiber diet. J. Nutr..

[20] Libiad M (2019). Hydrogen sulfide perturbs mitochondrial bioenergetics and triggers metabolic reprogramming in colon cells. J. Biol. Chem..

[21] FAO. Meat market review: Overview of global meat market developments in 2020. (2021).

[22] Ziegler A, Gonzalez L, Blikslager A (2016). Large animal models: The key to translational discovery in digestive disease research. Cell. Mol. Gastroenterol. Hepatol..

[23] Zhang Q, Widmer G, Tzipori S (2013). A pig model of the human gastrointestinal tract. Gut Microbes.

[24] Gonzalez LM, Moeser AJ, Blikslager AT (2015). Porcine models of digestive disease: The future of large animal translational research. Transl. Res. J. Lab. Clin. Med..

[25] Heinritz SN, Mosenthin R, Weiss E (2013). Use of pigs as a potential model for research into dietary modulation of the human gut microbiota. Nutr. Res. Rev..

[26] Sciascia Q, Daş G, Metges CC (2016). REVIEW: The pig as a model for humans: Effects of nutritional factors on intestinal function and health. J. Anim. Sci..

[27] Roediger WE, Truelove SC (1979). Method of preparing isolated colonic epithelial cells (colonocytes) for metabolic studies. Gut.

[28] Takano M, Yumoto R, Murakami T (2006). Expression and function of efflux drug transporters in the intestine. Pharmacol. Ther..

[29] Janssen JJE (2021). Novel standardized method for extracellular flux analysis of oxidative and glycolytic metabolism in peripheral blood mononuclear cells. Sci. Rep..

[30] Wüst RCI, Houtkooper RH, Auwerx J (2020). Confounding factors from inducible systems for spatiotemporal gene expression regulation. J. Cell Biol..

[31] Ryu AH, Eckalbar WL, Kreimer A, Yosef N, Ahituv N (2017). Use antibiotics in cell culture with caution: Genome-wide identification of antibiotic-induced changes in gene expression and regulation. Sci. Rep..

[32] Herst PM, Tan AS, Scarlett D-JG, Berridge MV (2004). Cell surface oxygen consumption by mitochondrial gene knockout cells. Biochim. Biophys. Acta.

[33] Pagliarani A, Nesci S, Ventrella V (2013). Modifiers of the oligomycin sensitivity of the mitochondrial F1F0-ATPase. Mitochondrion.

[34] Breen GA, Miller DL, Holmans PL, Welch G (1986). Mitochondrial DNA of two independent oligomycin-resistant Chinese hamster ovary cell lines contains a single nucleotide change in the ATPase 6 gene. J. Biol. Chem..

[35] Ramió-Lluch L (2014). Oligomycin A-induced inhibition of mitochondrial ATP-synthase activity suppresses boar sperm motility and *in vitro* capacitation achievement without modifying overall sperm energy levels. Reprod. Fertil. Dev..

[36] Yang H, Wang X, Xiong X, Yin Y (2016). Energy metabolism in intestinal epithelial cells during maturation along the crypt-villus axis. Sci. Rep..

[37] Lindeboom RG (2018). Integrative multi-omics analysis of intestinal organoid differentiation. Mol. Syst. Biol..

[38] Yang M (2021). Inhibition of mitochondrial function by metformin increases glucose uptake, glycolysis and GDF-15 release from intestinal cells. Sci. Rep..

[39] Yadav V, Talwar P (2019). Repositioning of fluoroquinolones from antibiotic to anti-cancer agents: An underestimated truth. Biomed. Pharmacother..

[40] Abad E (2019). Common metabolic pathways implicated in resistance to chemotherapy point to a key mitochondrial role in breast cancer. Mol. Cell. Proteomics.

[41] Morgun A (2015). Uncovering effects of antibiotics on the host and microbiota using transkingdom gene networks. Gut.

[42] Kalghatgi S (2013). Bactericidal antibiotics induce mitochondrial dysfunction and oxidative damage in mammalian cells. Sci. Transl. Med..

[43] Esner M, Graifer D, Lleonart ME, Lyakhovich A (2017). Targeting cancer cells through antibiotics-induced mitochondrial dysfunction requires autophagy inhibition. Cancer Lett..

[44] Chougule P (2012). Isolation and characterization of human primary enterocytes from small intestine using a novel method. Scand. J. Gastroenterol..

[45] Berger E (2016). Mitochondrial function controls intestinal epithelial stemness and proliferation. Nat. Commun..

[46] Salim AA (2016). Oligomycins as inhibitors of K-Ras plasma membrane localisation. Org. Biomol. Chem..

[47] Slott EF, Shade RO, Lansman RA (1983). Sequence analysis of mitochondrial DNA in a mouse cell line resistant to chloramphenicol and oligomycin. Mol. Cell. Biol..

[48] Tan B, Xiao H, Li F, Zeng L, Yin Y (2015). The profiles of mitochondrial respiration and glycolysis using extracellular flux analysis in porcine enterocyte IPEC-J2. Anim. Nutr..

[49] Fan Y-Y (2015). A bioassay to measure energy metabolism in mouse colonic crypts, organoids, and sorted stem cells. Am. J. Physiol. Gastrointestinal Liver Physiol..

[50] Pau G, Fuchs F, Sklyar O, Boutros M, Huber W (2010). EBImage–an R package for image processing with applications to cellular phenotypes. Bioinformatics (Oxford, England).

