# Supplementary material for: Mitochondrial and glycolytic extracellular flux analysis optimization for isolated pig intestinal epithelial cells
Source: Sci Rep. 2021 Oct 7;11:19961. doi: 10.1038/s41598-021-99460-0 (PMC8497502; doi:10.1038/s41598-021-99460-0)
Supplement: Supplementary file 1 — Supplementary Information. [file 41598_2021_99460_MOESM1_ESM.pdf]

Supplementary information for

Mitochondrial and glycolytic extracellular flux analysis optimization for isolated pig intestinal epithelial cells

A.F. Bekebrede[1,2], J. Keijer[1], W. J.J. Gerrits[2], V. C. J. de Boer[1*]

1. Human and Animal Physiology, Wageningen University and Research, 6708 PB WD Wageningen, The Netherlands; anna.bekebrede@wur.nl; jaap.keijer@wur.nl; Vincent.deboer@wur.nl

2. Animal Nutrition Group, Wageningen University and Research, 6708 PB WD Wageningen, The Netherlands; walter.gerrits@wur.nl;

[*]Corresponding author

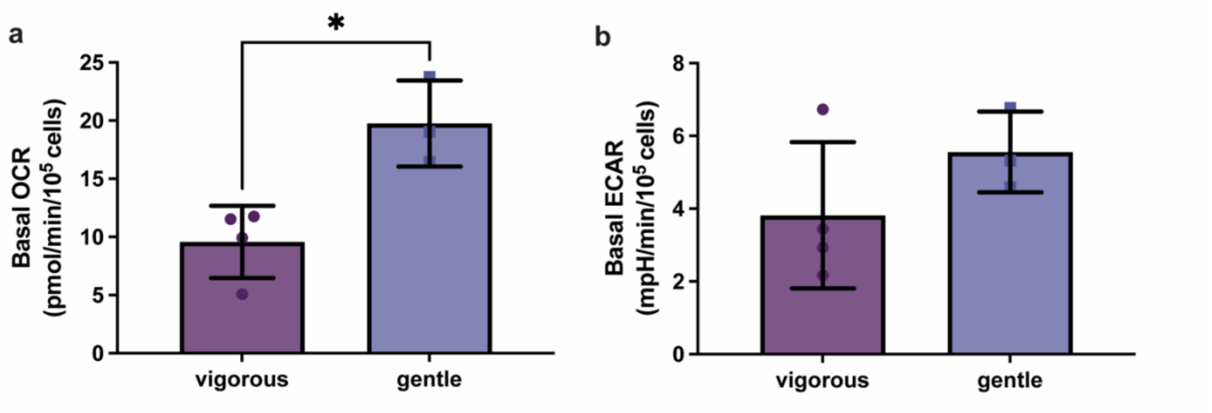

Supplementary figure S1 **Comparison of metabolic function following non-enzymatic enterocyte isolation methods.** (a) Basal OCR and (b) basal ECAR of colonocytes isolated with the vigorous or gentle methods (n=4 for vigorous method and n=3 for gentle method). Student's t-tests were performed to compare metabolic parameters of the vigorous and gentle methods, * indicates a p-value of ≤ 0.05.

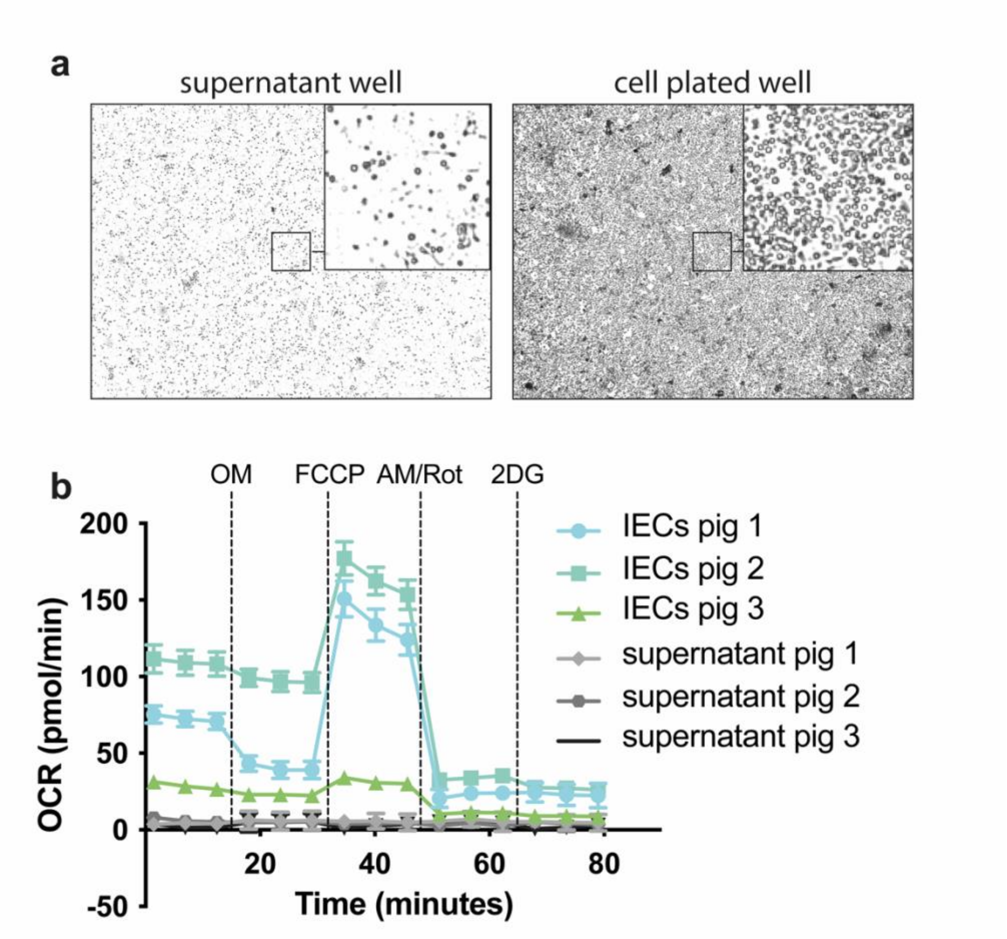

Supplementary figure S2 **Comparison of respiration of supernatant and IECs to rule out bacterial contamination.** (a) representative image showing a well where only supernatant was plated ('supernatant well') and a well where cell suspension was plated ('cell plated well'). Some cells are present in the supernatant plated well. (b) OCR time-course graph of supernatant compared to IEC plated wells.

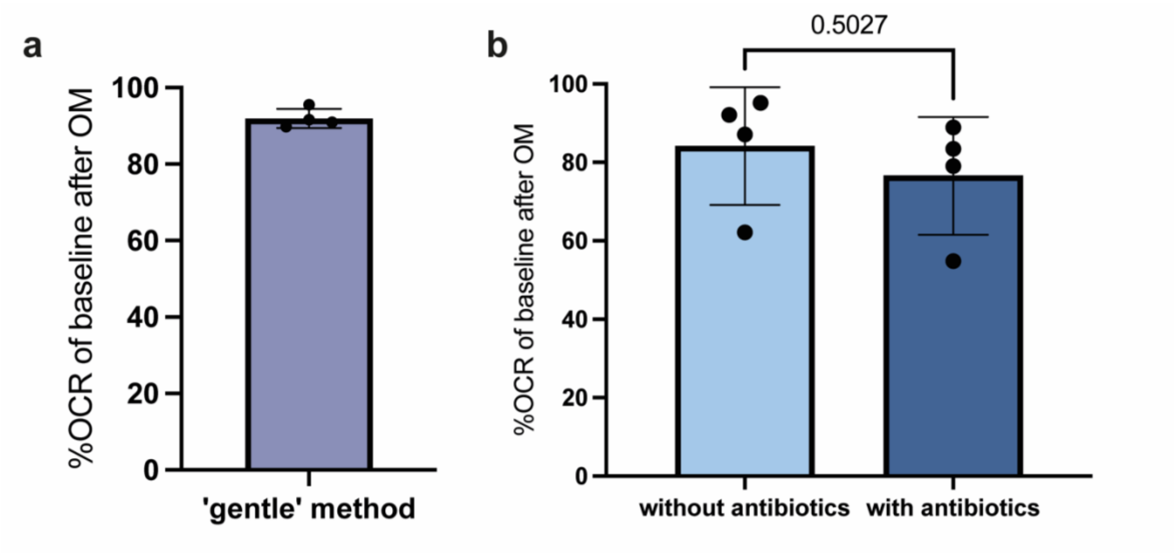

Supplementary figure S3 **Effect of Oligomycin IECs isolated using non-enzyme method and in the presence of antibiotics.** (a) OCR plotted as a % of basal OCR after Oligomycin (OM) injection for cells isolated using the non-enzymatic 'gentle' method. (b) OCR plotted as a % of basal OCR after Oligomycin injection for cells isolated in the absence (without) or presence (with) antibiotics. Student's t-tests were performed to compare OCR response in the presence or absence of antibiotics, p-value is shown in the graph.

Supplementary protocol for pig primary intestinal epithelial cells isolation and metabolic analysis

Isolation procedure

1) Prepare in advance:
   a) Modified Krebs Henseleit buffer (KHB; #K3753, Sigma Aldrich), according to manufacturer's protocol and set to pH 7.4. Can be stored at 4 °C for 2 weeks.
   b) Modified KHB buffer (#K3753, Sigma Aldrich), containing 2.5 g/L bovine serum albumin (BSA: #A7906, Sigma-Aldrich) and set to pH 7.4. Prepare on the day prior to the assay and store at 4 °C until use.
   c) Ca^{2+}-free KHB (118 mM NaCl, 4.7 mM KCl, 1.2 mM $MgSO_4$, 1.2 KH_2PO_4, 10 mM 4-(2-hydroxyethyl)-1-piperazineethanesulfonic acid (HEPES)) with 20 mM Ethylenediaminetetra-aceticacetic acid (EDTA) and 10 mM Dithiothreitol (DTT) and set to pH 7.4. Preferably make fresh on the day of assay, but no more than one day ahead.
   d) Ca^{2+}-free KHB (118 mM NaCl, 4.7 mM KCl, 1.2 mM $MgSO_4$, 1.2 KH_2PO_4, 10 mM HEPES) with 10 mM DTT and 400 U/mL hyalurondisase type IV (#3884, Sigma-Aldrich) and set to pH 7.4. Preferably make fresh on the day of assay, but no more than one day ahead.
   e) Pre-coat Seahorse XF 96 well assay plate with CellTak (#354240, Corning, New York, USA) according to manufacturer's protocol. Seal the places and store at 4 °C until use, but no more than one week in advance. On the morning of the assay, plate at room temperature (RT).
   f) Oxygenate the buffers prior to use with pure O_2.
2) Kill the animal and remove the intestine.
3) Separate the intestine and determine where the desired segment of intestine is located. For us, this was at 80% of the colon (from proximal).
4) Remove a ± 20 cm tissue piece and place in oxygenated modified KHB buffer containing 2.5 g/L BSA.
5) Once all the intestines are removed, flush the intestines using modified KHB buffer. We recommend to include no more than 8 animals per isolation round. This allows for fast isolation and fast metabolic analysis, in addition to allowing for sufficient replicates on the Seahorse XF assay plate.
6) Invert the intestines, using for example a long crochet hook. Make sure to remove any mesentery or fat tissue left on the segment, to allow for quick reversion in subsequent steps.

7) Clamp off the ends of the segments and fill the intestines with modified KHB buffer. Use a 50 mL syringe and standardize the amount of buffer between samples. With a 12-week-old pig we used 50 mL for a 20 cm long segment of the colon. The intestine should be somewhat distended to increase surface contact between the buffer and the intestinal mucosa.
8) Place the inverted segment in an Erlenmeyer with screwcap, containing about 150 mL Ca^{2+}-free KHB with 20 mM EDTA, 10 mM DTT and 2.5 g/L BSA.
9) Incubate for 20 min at 37 °C in a shaking water bath.
10) Pour away the buffer and refill the Erlenmeyer with another 150 mL of new Ca^{2+}-free KHB with 20 mM EDTA, 10 mM DTT and 2.5 g/L BSA.
11) Incubate for 20 min at 37 °C in a shaking water bath
12) Revert the intestines and fill them with Ca^{2+}-free KHB with 10 mM DTT and 400 U/mL hyaluronidase type IV (#3884, Sigma-Aldrich). Use about the same amount as previously, making sure the intestine is slightly distended to increase surface contact area between buffer and mucosa.
13) Place the intestinal segments in an Erlenmeyer containing 150 mL modified KHB and incubate for 15 min at 37 °C in a shaking water bath.
14) Gently massage the intestines for about 15 seconds and collect the contents in a conical 50 mL falcon tube.
15) Using a 70 µm cell strainer, filter the cells and place in a new 50 mL falcon tube
16) Spin the cells down for 5 min at 400 *x g*.
17) Discard the supernatant, pour about 10 mL modified KHB buffer containing 2.5 g/L BSA and shake the pellet loose.
18) Spin the cells down for 5 min at 400 *x g*.
19) Discard the supernatant, wash twice with 10 mL pH buffered Seahorse XF assay medium (#103575-100, Agilent Technologies) supplemented with 10 mM pH balanced glucose (#103577-100, Agilent Technologies), 2 mM pH balanced glutamine (#103579-100, Agilent Technologies), and 1 mM pH balanced pyruvate (#103578-100, Agilent Technologies).
20) Count the cells using the Bürker chamber.
21) Simultaneously measure viability with the K4 (Nexcelom biosciences) using ViaStain (#CS2-0106, Nexcelom Bioscience).
22) Prepare cell suspensions, seeding cells at 100,000 cells/well in 80 µL in a CellTak coated Seahorse XF 96 well assay plate.
23) Leave the cells to settle at RT for 10 minutes.
24) Spin down for 1 min at 200 G with zero break.

Imaging

25) Take Brightfield images of the inner probe area of each well in the XF96 cell plates using a 37 °C equilibrated Cytation 1 Cell Imaging Multi-Mode Reader (BioTek, Winooski, Vermont, USA) with a 4x objective. Set the LED intensity to 5 and integration time to 80 milliseconds. Use the 'User trained autofocus' option to obtain the best image quality.
26) After images are obtained, add another 100 µL of supplemented pH buffered Seahorse XF assay medium and place the plate into a non-CO_2 37 °C incubator.

Seahorse XF assay

27) On the day prior to the assay, equilibrate the Seahorse XF analyzer to 37 °C
28) Hydrate a cartridge overnight in a non-CO_2 37 °C incubator, using 200 µL sterilized milli-Q water (MQ) per well.
29) Replate MQ for XF Calibrant no less than 1 hour prior to the assay and incubate in a non-CO_2 37 °C incubator.
30) Prepare 10x injections in supplemented pH buffered Seahorse XF assay medium and pipet into the Seahorse cartridge:

Injection A (20 µL): 15 µM Oligomycin (#O4875, Sigma-Aldrich)
Injection B (22 µL): 10 µM carbonyl cyanide-p-trifluoromethoxyphenylhydrazone (FCCP; #C2920, Sigma-Aldrich
Injection C (25 µL): 12.5 µM Rotenone (#R8875, Sigma-Aldrich) and 25 µM Antimycin A (#A8674, Sigma-Aldrich)
Injection D (28 µL): 500 mM 2-deoxyglucose (2-DG; #D8375, Sigma-Aldrich)

31) Run Seahorse protocol. Include an equilibration step, and measure 3 times for 3 minutes, with 2 minutes of mixing between each measurement. Then, inject port A, and mix and measure another 3 times. Subsequently inject all other ports, always measuring and mixing 3 times prior to the net injection.
32) Optional: After the Seahorse XF assay run, image the plate in a similar manner as previously described, using the "user trained auto-focus" method to obtain the best quality images. This step can be included to check the amount of cell detachment.
